# Implementation of an Anterior Mediastinal Mass Pathway to Improve Time to Biopsy and Multidisciplinary Communication

**DOI:** 10.1097/pq9.0000000000000715

**Published:** 2024-02-05

**Authors:** Rachel E. Gahagen, William C. Gaylord, Meghan D. Drayton Jackson, Anne E. McCallister, Riad Lutfi, Jennifer A. Belsky

**Affiliations:** From the * Division of Pediatric Critical Care Riley Hospital for Children; † Department of Pediatrics, Indiana University School of Medicine; ‡ Division of Pediatric Hematology-Oncology Riley Hospital for Children.

## Abstract

**Background:**

Mediastinal masses in children with cancer present unique challenges, including the risk of respiratory and hemodynamic compromise due to the complex anatomy of the mediastinum. Multidisciplinary communication is often a challenge in the management of these patients. After a series of patients with mediastinal masses were admitted to Riley Hospital for Children Pediatric Intensive Care Unit, the time from presentation to biopsy and pathology was greater than expected. We aimed to reduce the time to biopsy by 25% and demonstrate improved multidisciplinary communication within 6 months of protocol implementation for patients presenting to Riley Hospital for Children Emergency Department with an anterior mediastinal mass.

**Methods:**

Quality improvement methodology created a pathway that included early multidisciplinary communication. The pathway includes communication between the emergency department and multiple surgical and medical teams via a HIPPA-compliant texting platform. Based on patient stability, imaging findings, and sedation risks, the approach and timing of the biopsy were determined.

**Results:**

The pathway has been used 20 times to date. We successfully reduced the time to biopsy by 38%, from 25.1 hours to 15.4 hours. There was no statistically significant reduction in time to pathology. The multidisciplinary team reported improved communication from a baseline Likert score of 3.24 to 4.

**Conclusions:**

By initiating early multidisciplinary communication, we reduced the time to biopsy and pathology results, improving care for our patients presenting with anterior mediastinal masses.

## INTRODUCTION

The American Cancer Society expects 15,190 new pediatric oncology diagnoses in 2023, including common malignancies such as lymphomas, T-cell leukemias, and germ cell tumors that often present with a large anterior mediastinal mass.^1–3^ The complex anatomy of the mediastinum makes these cases particularly challenging, as masses may compress the heart, large vessels, and bronchioles, potentially leading to acute respiratory and hemodynamic compromise.^4^ Patients are particularly high risk if they present with evidence of pericardial tamponade, cardiac dysfunction, tracheal compression, or a mediastinal mass ratio >0.45, as defined as the ratio of maximal transverse mass diameter to the thoracic diameter at T5-T6.^5^ If large masses compromise the great vessels or airway, patients risk becoming unstable. They may require emergent treatment with steroids, chemotherapy, or radiation therapy. Pretreatment of malignancies with steroids before biopsy has been shown to contribute to diagnostic uncertainty, and in some clinical trials, pretreatment assigns patients to higher-intensity regimens.^6–9^

Managing patients with anterior mediastinal masses can be challenging and requires coordination among multiple teams, including oncology, pediatric general surgery (PGS), anesthesia, interventional radiology (IR), and pediatric intensive care (PICU).^10–12^ Communication can be challenging, with multiple specialists needing to be contacted several times to coordinate care for these high-risk patients. Literature has emphasized that a multidisciplinary approach is vital for pediatric, adolescent, and young adult patients with anterior mediastinal masses.^13–15^ Institutions have implemented phone trees or conference calls to discuss these patients during presentations with intermediate success.

At Riley Hospital for Children (RH), all patients with new anterior mediastinal masses are routed through our emergency department (ED). Providers from the ED, PICU, and pediatric oncology departments are responsible for determining the patient’s disposition and coordinating biopsies with multiple subspecialists, including PGS, anesthesia, and IR. Previously, many individual conversations were required with the oncology or critical care teams, relaying recommendations of multiple teams via phone calls or messages, referred to as “middleman” communication.

By streamlining communication between teams with a new mediastinal mass pathway (MMP), we hypothesized that we could reduce the time to biopsy for children and AYAs with a new anterior mediastinal mass presenting to RH. Our primary smart aim was to reduce the time to biopsy from a mean of 25.1 hours by 25% within 6 months of protocol implementation for patients presenting to the institution with anterior mediastinal masses.^16–18^

A secondary aim for the pathway was to improve provider communication between multiple subspecialists via initiating an MMP and the use of Diagnotes (IUHealth, Indianapolis), a HIPAA-compliant text messaging platform. Secure texting applications have increased physicians’ perceived efficiency and satisfaction with communication with others.^19,20^ Using the group messaging feature and standardizing communication between teams, we hypothesized that we could improve communication and provider satisfaction between teams in 6 months, as measured by a Likert scale.

## METHODS

**Fig. 1.**
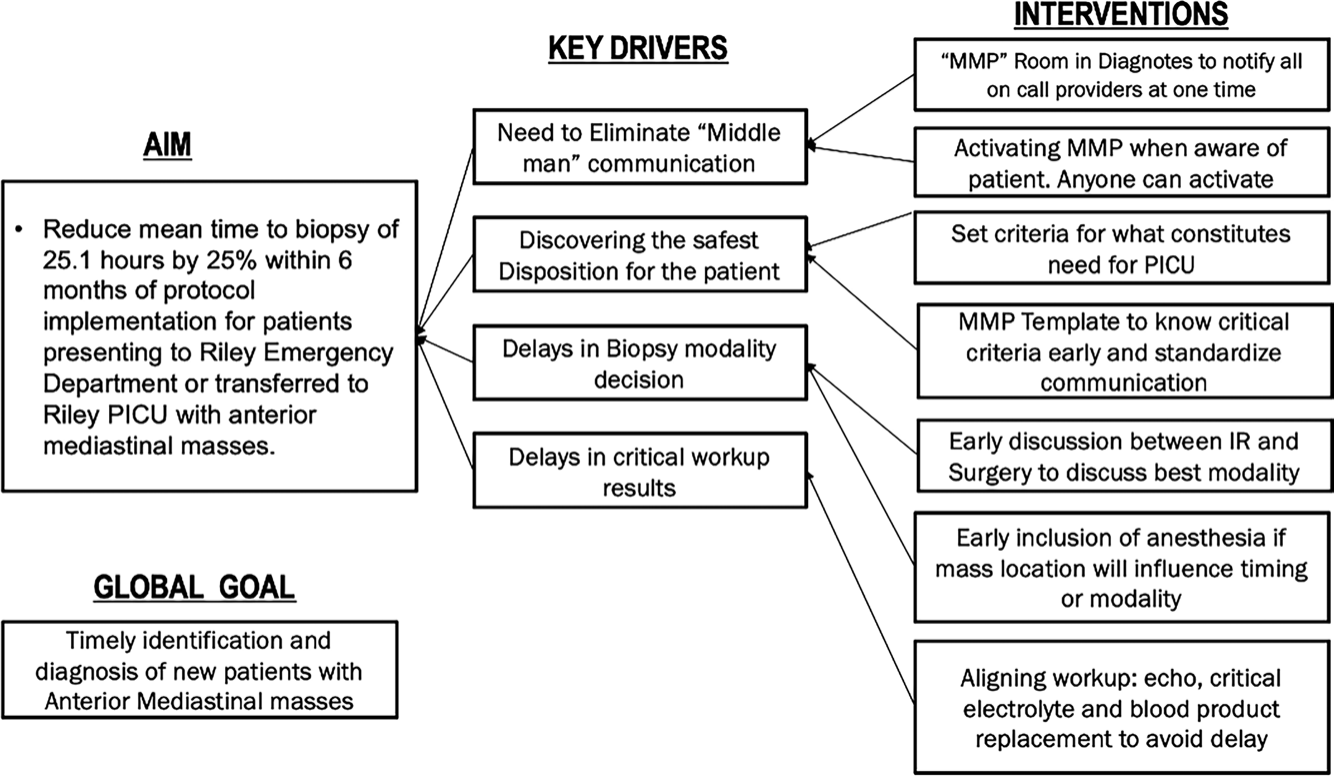
Key driver diagram identifying issues leading to delays in time to biopsy for patients presenting to this institution with anterior mediastinal masses.

The Indiana University institutional review board determined this project was exempt from full review. Baseline data were extracted from the electronic medical record (EMR) from May 2017 to June 2021. We included patients with an anterior mediastinal mass on imaging, aged 0–25 years, admitted to the inpatient floor or ICU for an initial diagnostic workup at RH. Provider presurveys were distributed between August 2021 and September 2021 to a group of subspecialties, including oncology, PGS, IR, and PICU. Questions focused on communication between teams before implementing the pathway. Data were entered and stored in a Research Electronic Data Capture (REDCap).^21^ The IHI Model for Improvement was used for project design, development of a key driver diagram, and implementation (Fig. 1). Data were analyzed, and charts were created using quality improvement Macros.

### MMP Survey Design

**Fig. 2.**
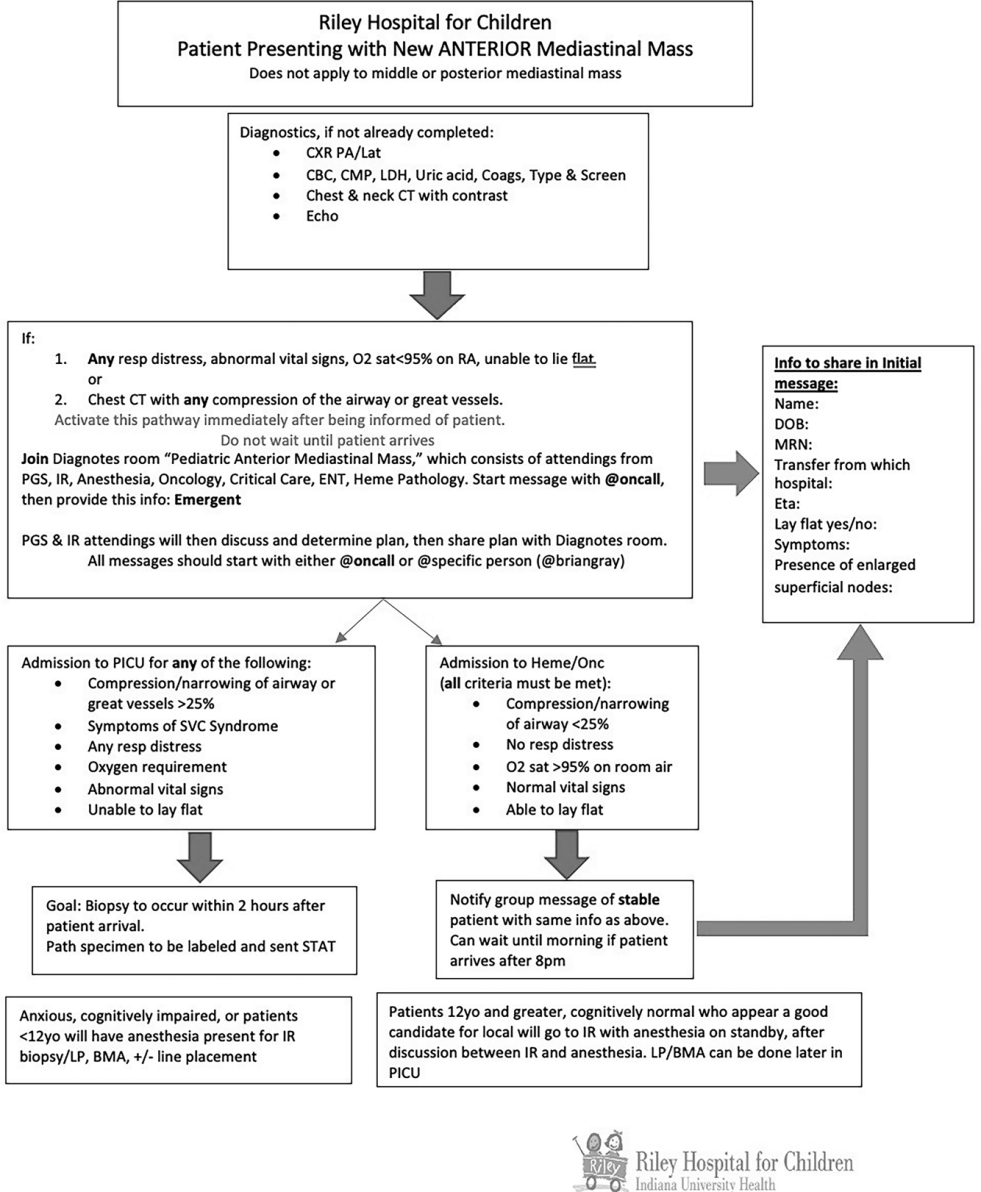
MMP flowsheet.

Key drivers identified at this institution for delay of care of new mediastinal mass presentation include: “middleman” communication, unclear disposition for the patient, delays in biopsy decision-making, and delays in pathology diagnosis (Fig. 1). Pre-survey questions identified “middleman” communication, biopsy modality, anesthesia safety, and disposition to the oncology floor or PICU as notable delay causes. Using these initial responses, we developed an MMP, which was implemented in September 2021 (Fig. 2).

Following each MMP activation, all team members involved in the initial activation message received an anonymous postactivation survey with questions assessing satisfaction with communication between teams, ease and appropriate use of the secure messaging platform, any delays in patient care, and directly soliciting feedback on the pathway (**See figure 1, Supplemental Digital Content**, which shows postactivation survey. http://links.lww.com/PQ9/A539.) Surveys were distributed within 10 days of pathway activation to limit the risk of recall bias.

The pathway has undergone three Plan-Do-Study-Act (PDSA) cycles (Table 1). After each PDSA cycle, the multidisciplinary team reviewed the pathway again, and feedback on potential changes was sought. A champion for each involved division would educate their respective division on any changes made.

**Table 1.** PDSA Cycles Summary

PDSA Cycle	Intervention	Time Frame
PDSA 1	Implementation of the Mediastinal Mass Pathway	September 2021–October 2021
PDSA 2	Re-education of emergency room providers regarding activation criteria	November 2021–May 2022
PDSA 3	Decision regarding pathway activation to be determined by on-call oncologist	June 2022–June 2023

PDSA cycle 1 started in September 2021 with the implementation of the pathway after integrating the secure messaging system with the on-call provider schedules. PDSA cycle 2 started in November 2021 and focused on the re-education of our ED providers, as they were often the initial team activating the pathway and expressed uncertainty with initial pathway activation criteria. PDSA cycle 3 began in April 2022. During this cycle, oncology fellows or attendings activated the pathway. ED providers had variable experiences with the pathway due to increased turnover, resulting in increased inappropriate activations.

### HIPPA-compliant Messaging (Diagnotes)

Diagnotes is a HIPAA-compliant secure messaging platform utilized by RH since 2019. A key feature of the platform is its ability to utilize group messaging and link to teams on-call. Once a patient was found to have an anterior mediastinal mass, the MMP was activated via Diagnotes by an MMP team member upon notification of a patient presenting with an anterior mediastinal mass. Once activated, Diagnotes automatically pulls in the physician on call for each multidisciplinary team service into the MMP group message. This platform allowed discussion of the patient’s findings and vitals and planning for the biopsy to occur immediately and simultaneously between all multidisciplinary team members. This eliminated the “middleman” communication and allowed teams to relay concerns in real-time. Any updates could be shared as the patient arrived or if there were changes in the patient’s condition.

### MMP Communication

Based on the criteria laid out in the pathway, oncology and PICU teams would discuss disposition to the oncology unit or PICU. The pathway defined a set of criteria for final disposition, including tracheal or great vessel compression >25%, supplemental oxygen requirement, inability to lay flat, symptoms of superior vena cava syndrome, or abnormal vital signs. Surgical and IR physicians reviewed imaging and determined whether an IR or surgical approach would be safest. Anesthesia reviewed imaging and vital signs to determine risks and assess airway concerns. Communication was initially done through text messages. However, when patients were at particularly high risk for decompensation or were concerned about the potential need for ECMO, bedside multidisciplinary discussion could be organized via texting. No misinterpretation occurred with the texting modality.

### Measures

We aimed to reduce the time to biopsy from a mean of 25.1 hours by 25% within 6 months of protocol implementation. The pathway (Fig. 2) was designed to address many issues identified in the key driver diagram. Time to biopsy was chosen as the primary outcome for the patients included in the pathway. Time to biopsy was defined as the time of the “admit to inpatient” order in the EMR (zero timepoint) to the procedure start time listed in the operative note. Institutional baseline review showed a mean time of 25.1 hours from presentation to biopsy. Time to final pathology was measured from the time to biopsy until the finalized pathology report, with the institutional mean being 74 hours at baseline. Finalized pathology is often required for most research trial enrollment and selecting targeted treatment plans not involving a clinical trial.

A secondary aim of the pathway was to improve communication between providers as measured on a Likert scale. The multidisciplinary approach to these patients requires effective communication and was a common complaint in analyzing the baseline surveys. Baseline surveys were distributed to members of each specialty, analyzing several aspects of the communications between the primary team and subspecialists in the workup and care of these patients.

Process measures included identifying patients with a new anterior mediastinal mass diagnosis without protocol initiation. Activations of the pathway for patients who did not meet the criteria were also tracked. Missed and inappropriate activations were chosen as balancing measures.

## RESULTS

**Fig. 3.**
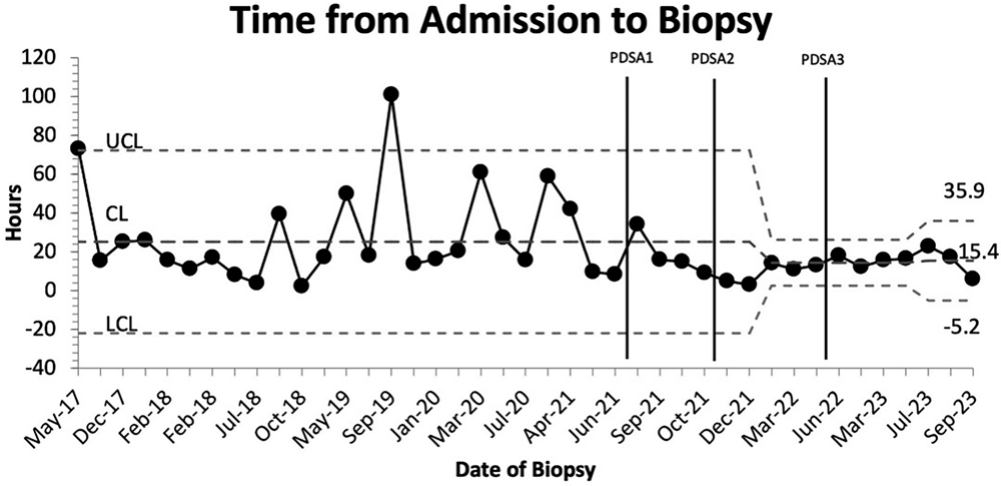
Control chart showing baseline through PDSA 3 of time elapsed from pathway activation until the start of biopsy.

The baseline cohort included 29 patients identified from 2017 to 2021 with anterior mediastinal masses that would meet the criteria for pathway activation. After the pathway was implemented, the pathway was activated twenty times from September 2021 until September 2023. There were zero missed activations. Five of these activations were inappropriate. Of these inappropriate activations, one patient had prominent hilar nodes and no true mediastinal mass, two patients had posterior mediastinal masses, and two patients without mediastinal masses. Still, the pathway was used to coordinate other oncology biopsies. Fifteen patients met the activation criteria. Fourteen of the 15 patients (94%) had imaging showing an anterior mediastinal mass before arrival at our institution. A control chart showed the time to biopsy for patients presenting with an anterior mediastinal mass and demonstrated the institutional baseline through PDSA cycle 3 (Fig. 3). There is reduced variability in time to biopsy, as seen in Figure 3. Following pathway implementation at the end of PDSA 3, there was a 38% reduction in the meantime-to-biopsy for patients from baseline from 25.1 to 15.4 hours (range 3–18 hours). The mean time to final pathology diagnosis was not statistically significant. Figure 3 reveals baseline data. There were two outliers above the upper control line: one patient who was seen and then discharged for outpatient imaging and IR biopsy and another patient who was admitted to the hospitalist service for upper respiratory illness and presyncopal symptoms who was later found to have a mediastinal mass on day 3 of admission. Eight patients underwent biopsy with general surgery in the OR, and seven patients underwent biopsy by IR. No statistical difference was found between the time to biopsy and the method or service obtaining the biopsy. No patients required treatment with steroids or chemotherapy before the biopsy was obtained.

**Fig. 4.**
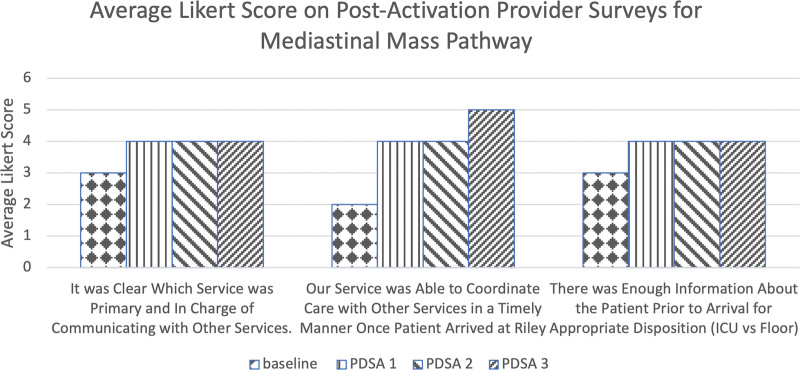
Average Likert score from postactivation provider surveys. Response: 1 = strongly disagree; 2 = slightly disagree; 3 = neither agree nor disagree; 4 = slightly agree; 5 = strongly agree.

Figure 4 shows the results of the postactivation surveys distributed to providers. The greatest score improvement was in response to the statement, “Our service was able to coordinate care with other services promptly once the patient arrived.” The average response increased from “slightly disagree” to “strongly agree” through PDSA 3. Through PDSA cycle 3, for statements “There was enough information about the patient before arrival for appropriate disposition” and “It was clear which oversaw the workup and communication with other services,” response increased from “neither agree nor disagree” to “slightly agree” (Fig. 4). The postactivation surveys revealed active providers perceived the pathway to improve several aspects of multidisciplinary communication, but further improvements are being sought regarding appropriate communication. The number of those who strongly agree with being satisfied decreased after PDSA cycle 3. After discussion with each division champion, inappropriate activations were a point of dissatisfaction. Re-education was necessary for all divisions on when and how to activate the pathway.

## DISCUSSION

Proper management of patients with anterior mediastinal masses, particularly the importance of a multidisciplinary approach, is critical for patient safety.^2,4,5,13–18,22–28^ Numerous studies have highlighted the challenges in communication between teams.^2,4,13,15–18,22,27–30^

Despite common challenges, including patient nothing-by-mouth status, OR, and provider availability, which will affect biopsy times, our results demonstrate the pathway’s successful ability to respond urgently to the needs of the patient.

A 38% reduction in time to biopsy was achieved at the end of PDSA cycle 3. Fleming et al reported a 50% reduction in time to biopsy implementing their MMP using a conference call/phone tree system.^16^ The protocol initiated at our institution decreased the time to biopsy after 6 months of implementation, whereas Fleming et al implemented it over 10 years. Our protocol also had a mean time to biopsy of 15 hours compared with the 24 hours reported in the study by Fleming et al.

One additional specialty added to the pathway, not included in previous studies, was a hematology pathologist. This ensured the pathologist was prepared to accept the sample and prepare for flow cytometry and morphology. One biopsy sample in PDSA cycle 2 was a suspected mediastinal teratoma, which at RH is received by our solid tumor pathology department. This sample required a more advanced pathology workup, leading to a longer time to diagnosis.

We had no missed activations of the protocol, indicating high compliance. During PDSA 1 and 2, there were five inappropriate activations for patients who did not meet the criteria for activation. In discussion with the ED providers who activated the protocol, they did not feel they adequately understood the criteria for activation. This knowledge deficit formed the basis for intervention for PDSA cycle 3, in which we have shifted the onus of activating the protocol to the oncology team. Any provider who believes a patient qualifies for pathway activation would notify the oncology provider on-call to discuss. This change was well received, with no inappropriate activations, as reflected in the overall positive responses to our survey questions in PDSA cycle 3.

As with all studies, we identified potential challenges and limitations within our pathway. One challenge was determining time zero when considering time to biopsy. Although 93% of the patients were identified as having a mediastinal mass at outside institutions, it was difficult to determine a standard timepoint in the EMR to measure the elapsed time from initial patient notification at an outlying hospital to arrival and then to biopsy. Potential challenges include the availability of emergency medical services for transport, or the availability of the imagining required for diagnosis. Another limitation for potential delay was operating room availability. We chose the admission order placement as time zero to mitigate these challenges.

Finally, barriers were encountered as we implemented the pathway at a large tertiary academic pediatric hospital. Educating several departments of attending and fellow providers from oncology, PICU, ED, PGS, and IR on the pathway from PDSA cycles was challenging. We overcame this barrier by identifying an invested “champion” from each multidisciplinary team responsible for educating colleagues and relaying concerns regarding the pathway or activations.

## SUMMARY

Implementing an anterior MMP at RH reduced the mean time to biopsy by 38% for pediatric patients, with zero requiring pretreatment for airway compromise before biopsy. Furthermore, there was improved physician satisfaction with communication in a multidisciplinary team. As the medical community continues to use and integrate messaging platforms routinely into patient care, it is crucial to understand the successes and challenges identified here. With continued use and expansion to additional institutions, we can provide safe, effective, and rapid care for oncology patients with mediastinal masses.

## ACKNOWLEDGMENTS

The authors acknowledge Audrey Leisinger, RN, for assistance with this study.

## Supplementary Material


