# Supplementary material for: Implementation of an Anterior Mediastinal Mass Pathway to Improve Time to Biopsy and Multidisciplinary Communication
Source: Pediatr Qual Saf. 2024 Feb 5;9(1):e715. doi: 10.1097/pq9.0000000000000715 (PMC10843474; doi:10.1097/pq9.0000000000000715)
Supplement: Supplementary file 1 [file pqs-9-e715-s001.pdf]

Post-Activation Follow Up Survey

Please complete the survey below.

Thank you!

Today's Date

____

Were you involved in care recently of a patient with a suspected mediastinal mass?

- ○ Yes
- ○ No

What department are you with?

- ○ PICU
- ○ Heme/Onc
- ○ IR
- ○ General Surgery
- ○ ED
- ○ ENT
- ○ Pathology

If the patient came in overnight, when were subspecialists/other teams notified of the patient and possible need of their services?

- ○ Overnight/immediately on arrival
- ○ Overnight but First thing in the morning by day team
- ○ Overnight but after rounds
- ○ Daytime Arrival, immediately notifying teams
- ○ unsure

Was the Mediastinal Mass Pathway activated?

- ○ Yes
- ○ No
- ○ Unsure

When was your team notified about the patient?

- ○ Immediately via Diagnotes Room or By Primary team
- ○ First thing in the morning
- ○ After rounds

Was there a service that should have been involved earlier?

- ○ IR
- ○ Surgery
- ○ Pathology
- ○ PICU
- ○ Oncology
- ○ Other

If other, then who should have been involved

____

For the following statements, please select how strongly you agree, disagree. If you feel like the statement does not apply to you, select N/A

	Strongly Disagree	Slightly Disagree	Neither Agree nor Disagree	Slightly Agree	Strongly Agree	N/A
Our service received adequate communication about the patient prior to arrival at Riley.	○	○	○	○	○	○
